# Supplementary material for: Longitudinal Assessment of Tau-Associated Pathology by 18F-THK5351 PET Imaging: A Histological, Biochemical, and Behavioral Study
Source: Diagnostics (Basel). 2021 Oct 12;11(10):1874. doi: 10.3390/diagnostics11101874 (PMC8535097; doi:10.3390/diagnostics11101874)
Supplement: Supplementary file 1 [file diagnostics-11-01874-s001.zip › diagnostics-1332034-supplementary.pdf]

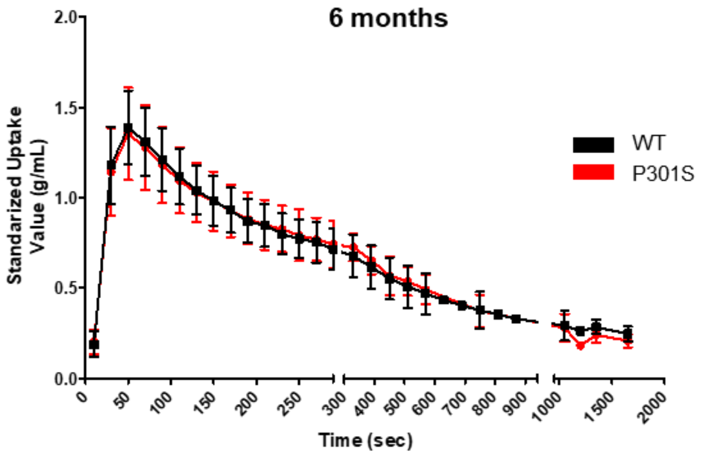

Supplementary Figure S1. Dynamic ^{18}F-THK5351 acquisition in P301S and WT mice. Initial probe standardization was performed using dynamic acquisition from 10 to 1800 s, every 20 s, right after administration of the probe in the tail vein of 6-month-old WT mice (black line) and P301S mice (red line). Measurement was performed in the whole brain. After initial uptake, SUV (g/mL) decayed and reached plateau after 15 min (900 s) acquisition. No statistical differences were found between WT and P301S animals (two-way ANOVA, $p > 0.05$).

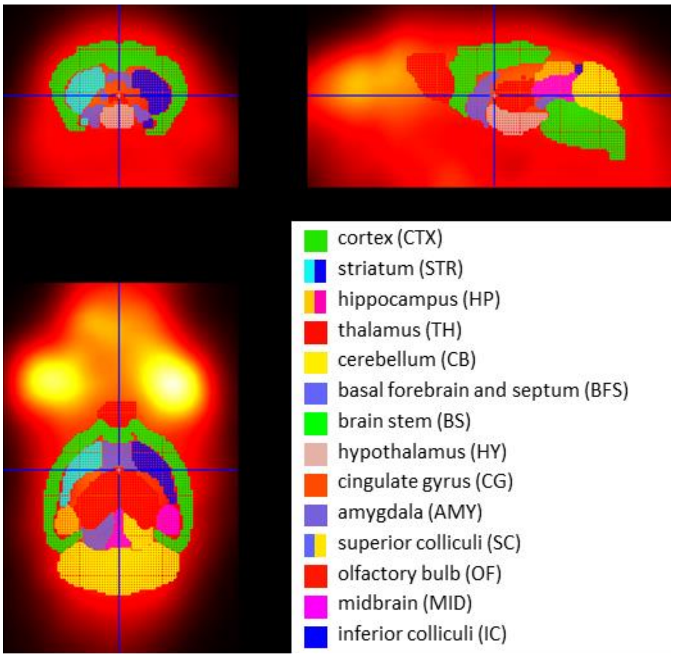

Supplementary Figure S2. PET analyzed mouse brain areas. Several brain regions of interest (ROI) were measured over the course of the scan. Representative images: coronal (top left), sagittal (top right), and longitudinal (bottom left) planes show selected brain areas that were analyzed after imaging by overlaying the mouse atlas fitted using CT images. Selected ROIs are striatum (STR), cortex (CTX), hippocampus (HP), thalamus (TH), cerebellum (CB), basal forebrain and septum (BFS), hypothalamus (HY), amygdala (AMY), brain stem (BS), cingulate gyrus (CG), superior colliculi (SC), olfactory bulb (OF), midbrain (MID), and inferior colliculi (IC).
